# Treatment Adherence in Patients with Lung Cancer from Prospects of Patients and Physicians

**DOI:** 10.31557/APJCP.2021.22.6.1891

**Published:** 2021-06

**Authors:** Kyriakos Souliotis, Lily E Peppou, Marina Economou, Androniki Marioli, Sofia Nikolaidi, Maria Saridi, Dimitrios Varvaras, Antonia Paschali, Konstantinos N Syrigos

**Affiliations:** 1 *Faculty of Social & Political Sciences, University of Peloponnese, Corinth, Greece. *; 2 *School of Nursing, University of Thessaly Greece. *; 3 *Community Mental Health Centre, University Mental Health Research Institute (UMHRI), Athens, Greece. *; 4 *First Department of Psychiatry, Aiginition Hospital, Medical School, Kapodistrian University of Athens, Athens, Greece. *; 5 *Division of Medical Oncology, Third Department of Medicine, Kapodistrian University of Athens, Athens, Greece. *; 6 *Health Policy Institute (HPI), Athens, Greece. *; 7 *Surgical Oncology Unit, Nuova Villa Claudia, Rome, Italy. *; 8 *Faculty of Nursing, School of Health Sciences, Department of Mental Health and Behavioral Sciences, Kapodistrian University of Athens, Athens, Greece. *

**Keywords:** Treatment adherence, lung cancer, patients’ and physicians’ prospects, depression, anxiety

## Abstract

**Purpose:**

Adherence to treatment can be defined as the degree to which a patient’s behavior is consonant with medical or health advice he or she receive as part of his treatment regimen. The aim of this study was: 1) to measure the rate of treatment adherence to among patients with lung cancer from the prospect of both patients and physicians, 2) to measure the degree of concordance between the two prospect, and 3) to identify factors related to adherence for both prospect (patients and physicians).

**Materials and Methods:**

A total of 250 patients were included in this study. Information about socio-economic characteristics, depressive and anxiety symptoms (Hospital Anxiety and Depression scale), nicotine dependence (Fagerstrom scale), barriers to accessing care, and the level of treatment adherence was collected through interview. Physicians were enquired about disease and treatment variables as well as patients’ level of adherence.

**Results:**

From the patient perspective, only 1.2% of patients displayed poor adherence; whereas the corresponding percentage among physicians was 12.4%. The concordance between the two was low: 0.244. The correlation of measurements made on the same individual was found to be equal to 0.14. Barriers to accessing medication (O.R.=2.82, 95% C.I.: 1.01-8.09) was the only risk factor when adherence was self-rated; barriers to accessing medication (O.R.=2.45, 95% C.I.: 1.03-5.86), education equal to 12 years (O.R.=0.33, 95% C.I.: 0.13-0.82) or higher than 12 years (O.R.=0.28, 95% C.I.: 0.08-0.96), nicotine dependence (O.R.=1.41, 95% C.I. 1.17-1.69) and HADS anxiety score (O.R.=1.15, 95% C.I. 1.03-1.30) were the predictors in physicians’ rating.

**Conclusions:**

Differences in rating adherence may underpin communication gaps between patients and physicians. Systemic determinants of poor adherence should not be overlooked. A concerted effort by researchers, physicians and policy makers in defining as well as communicating adherence, while removing its barriers should be made.

## Introduction

Adherence can be defined as the degree to which a patient’s behavior is consonant with medical or health advice (Haynes et al., 1979) and it constitutes an indispensable part of a successful health outcome (Claxton et al., 2001; DiMatteo et al., 2002). However, converging evidence indicates that approximately 40% of patients do not take prescribed medication at allor take it incorrectly, while twice that number fail to follow dietary and lifestyle advice, including dietary restrictions, increased exercising, and reduced smoking (Brownell and Cohen, 1995; Epstein and Cluss, 1982; Norman et al., 2000; Adnan et al., 2016). Non-adherence may mislead the clinician who may ascribe patients’ exacerbation to drug inactivity, resulting in redundant diagnostic testing, hospitalizations or/ changes in dosage or regimen (Avornet al., 1998). Low adherence rate has also been linked to heightened physician visits, elevated admission rates and longer hospitalizations (Greenberg, 1984; Lebovits et al., 1990). Additionally, suboptimal adherence may jeopardize the patient-physician alliance, as misconceptions about treatment effects may impinge on the communication between the two and alterpatients’ views about care (Waterhouse et al., 1993).

A way forward for improving adherence is identif 360 ying indicators and reasons for non-adherence. Nonetheless existing literature on determinants of adherence is scarce and largely inconsistent (Di Matteo et al., 2002; Partridge et al., 2002). Overall, non-adherence is complex and influenced by many factors: patient characteristics, aspects of the disease and the treatment regime as well as facets ofthe medical care system (Ruddy et al., 2009; Verbrugghe et al., 2013; Eldessouki et al., 2018). Specifically, socio-demographic characteristics, financial hardship, family instability, lack of social support, depression, treatment complexity and duration, elevated treatment costs, medication side- effects and poor therapeutic alliance have been found to predict non-adherence (Di Matteo et al., 2000; Golin et al., 2002; Ickovics and Meade, 2002; Krueger et al., 2003; Osterberg and Blaschke, 2005; Paranjpe et a., 2019). Further, from studies investigating patient reports, typical reasons include assigning low priority to medication, lack of appropriate information, emotional factors and unintentionally missing does due to oversleeping, forgetfulness, eating meal at a wrong time and social situation (Cramer, 1991; Walsh et al., 2001; Bassan et al., 2014; Lavdaniti et al., 2018). Based on the aforementioned, physicians may unintentionally promotenon-adherence by prescribing complex regimes; overlooking patients’ lifestyle and financial constrains; not explaining adequately the benefits of treatment and its side effects; and not working enough on building a good therapeutic alliance. 

Adherence in oncology is assumed to be optimum due to the severity of the disease and its dire implications (Osterbergand Blaschke, 2005; Haynes et al., 2002). There is dearth of research on the topic, with the vast majority of studies addressing breast cancer (Ruddy et al., 2009). For example, Bonadonna andValagusa (1981), have shown that breast cancer patients who received less than 85% of their adjunctive chemotherapy had shorter relapse free and total survival times as compared to breast cancer patients with better adherence levels. More recently, a retrospective study analyzing 3,976 patients first diagnosed with primary breast cancer demonstrated a significant association between treatment adherence and prolonged recurrence free and overall survival, with the greater the number of violations in guideline adherence, the lower the overall survival (Wockel et al., 2010). The US National Health and Wellness Survey (Goren and Di Bonaventura, 2013), reported that 71% of patients with melanoma, 65% of patients with lung cancer and 59% of patients with leukaemia displayed non-adherence with oral anticancer therapy. Younger people, unemployed individuals and heavy smokers showed increased likelihood of non-adherence. Moreover, non-adherence was associated with worse physical and mental health as well as increased hospitalizations and emergency hospital visits. Further, a similar research, explored reasons for non-adherence among patients with breast cancer with a special interest in patients’ beliefs. Their study demonstrated that beliefs about the necessity of medication and concerns about side effects are important issues to be considered by clinicians when they devise a treatment plan (Grunfeld et al., 2005).Therefore, even in the realm of oncology, non-adherence constitutes a vitally important target for intervention. 

In this context, the present study endeavored to investigate the following objectives:

• To assess the rate of treatment adherence from patients’ and physicians’ prospect

• To estimate the degree of concordance between the two prospects

• To identify risk factors contributing poor adherence from patients’ and physicians’ prospect

## Materials and Methods

*Sample*

A total of 250 adult patients with lung cancer, stage III and IV were consecutively recruited from the Oncology Unit of the Sotiria General Hospital. The particular unit is a specialized public health service provider for the treatment of various types of cancer, receiving patients from all parts of Greece. During its 10-year operation, the unit has treated more than 4500 patients with cancer. At the same time, the General Hospital of Sotiria is the only hospital in Greece with a long-standing specialization in respiratory diseases. Therefore, its oncology unit has a further specialization in lung cancer. In fact, the oncology unit of Sotiria is considered to be the main public health treatment provider for lung cancer in the country and a centre of excellence. The stage of cancer was in accordance with the Tumor Node Metastasis (TNM) Staging System of the American Joint Committee on Cancer (Amin, 2017).

Patients were excluded if they were not fluent in Greek or their health status was so severe that the interview could not have been conducted. Moreover, in cases where there had been metastasis of lung cancer in the brain, patients with substantial neurological deficits were excluded from the sample. All patients provided informed consent for their participation.

All patients who met eligibility criteria had the same probability to be enrolled in the study. Participants were selected at random from the hospital’s registry.

*Instrument*

The interview was conducted 3 months after patients started their treatment plan at the unit. It consisted of the following sections:

Demographic and socio-economic characteristics: Information was collected with respect to participants’ gender, age, nationality (Greek vs. non-Greek), place of residence (Athens vs. other city), family status, number of underage children, living arrangement (living alone-living with own family- living with other relatives), educational attainment, employment status, monthly individual income and public health insurance (yes – no).

The degree of financial hardship was assessed with the Index of Personal Economic Distress (Madianos et al., 2011). It encompasses 9 items enquiring about participants’ difficulty to meet regular financial demands of a household, such as public utilities, rent, super market and others, during the past 6 months. Each item is rated on a 3-point scale: 0= I do not have this type of expense, 1 = rarely, 2= sometimes and 3=often. Higher composite scores indicate higher degree of financial hardship. The index has displayed good psychometric properties (Madianos et al., 2011). 

*Depression and anxiety*

Clinical symptoms of depression and anxiety were measured with the Hospital Anxiety and Depression Scale, HADS (ZigmondandSnaith,1983). HADS is frequently employed in hospital settings and in cancer research due to addressing primarily the psychological- rather than the physical symptoms of depression and anxiety (e.g. insomnia). The scale consists of 7 items tapping clinical depression and 7 tapping anxiety. In each item, responses are made on a 4-point scale (0-3), with higher scores indicating greater symptom severity. The scale has been validated in Greece and it has displayed good psychometric properties (Michopoulos et al., 2007).

Nicotine dependence: Nicotine dependence was measured with the FagerstromTest for Nicotine Dependence. It entails 6 questions, whose ratings are summed up to produce a composite score. Scale scores can range from 0-10 with higher scores denoting more severe dependence. The scale has displayed very good psychometric properties(WHO, 2008). 

Barriers to treatment: Barriers to treatment were recorded by utilizing corresponding items of the Health Outcomes Patient Environment (HOPE) study(Souliotis et al., 2014). Specifically, participants were asked whether they had been experiencing obstacles in accessing medical/pharmaceutical care during the past year. In case of an affirmative answer, they were further asked to identify the obstacles they tackled.

*Disease and treatment characteristics*

Information was also gleaned with regard to disease and treatment characteristics: disease type (non-small cell lung cancer vs. small cell lung cancer), duration since the diagnosis, type and number of medications.

*Adherence*

Treatment adherence was assessed through a self-report question: “How would you rate your consistency in following your physician’s treatment recommendations in terms of pharmaceutical treatment, smoking dietary and lifestyle habits”.Responses were made on a 5-point scale ranging from “very bad” to “very good”. The question was based on work investigating medication adherence in HIV patients (Lu et al., 2008) and in light of evidence substantiating the use of self-report measures in treatment adherence (Hays et al., 1987; Di Matteo et al., 2003).

Concomitantly, a similar question was addressed to the treating physician.More specifically treatment physicians were asked to rate their patient’s adherence based on the item: “How do you rate patient’s consistency in following your recommendations (please take into account pharmaceutical treatment, smoking and dietary habits, exercise, etc.)?”. Responses were rated based on a 5-point Likert scale ranging from “very bad” to “very good”.

To increase inter-rater reliability among physicians, clinicians were instructed to think of the treatment plan in a holistic-integrative manner, in line with the service provision. They were instructed to take into account medication adherence, lifestyle changes, emergency hospital visits, the duration of hospitalizations, adverse events as well as missing appointments at the clinic. Many examples of patients’ adherence patterns were given to physicians in order to rate them during staff meetings. The research team made 2-3 meetings with the physicians in the hospital so as to i) ensure questionnaire’s content validity through physician’s feedback on it and ii) to ensure that all adherence patterns are rated by different physicians in the same manner.When their ratings started to converge (roughly after 17 examples of patients not included in this sample), calibration phase was ended. The first 50 cases of the sample were rated during staff meetings. 

Finally, participants’ affirmative response to the question “do you smoke during the last 12 months?” was considered to suggest suboptimal adherence.

Data were collected in the form of a face-to-face interview from January to December 2016.

*Procedure*

A total of 15 undergraduate and postgraduate students of social and health sciences received training on the epidemiology and treatment of cancer as well as on research and interviewing skills. Once students were considered ready for data collection, they visited the Oncology Unit of Sotiria General Hospital on a weekly basis and after agreement with physicians, they approached potential participants. After informed consent was obtained, the interview was initiated. Students received supervision by senior researchers twice a month. 

*Ethics approval and informed consent*

The research protocol had received approval from the Ethical Research Committee of the University of Peloponnese and the Ethics Committee of the Sotiria General Hospital, in accordance with the ethical standards delineated in 1964 Declaration of Helsinki. Ρrot. Number: 8/17.03.2015. All participants provided informed written informed consent for their participation.

*Statistical analysis*

All analyses were conducted using Stata version 12. Descriptive analyses were performed using percentages and absolute frequencies for categorical and ordinal variables, means and standard deviations were used for continuous variables which were found to be normally distributed while medians and ranges were used to describe non-normally distributed variables.

Intra class correlation coefficient was estimated using a two-way random-effects model, to assess the inter rater reliability between the two independent groups of raters, the patients themselves and their doctors. Intra class correlation coefficient was selected because it is an index that reflects both degree of correlation and agreement between measurements (Koo and Mae, 2016).

Univariate analyses were used to identify the demographic, socio-economic and clinical characteristics associated with the rate of adherence as stated by the patients themselves and their doctors at the p<0.10 level. All variables statistically significant at the p<0.10 level, entered in a multiple ordinal logistic model. As a result, two different multiple ordinal logistic regressions were conducted and both of them were found to satisfy the proportionality of odds test.

## Results

Sample characteristics are presents in Table 1. More than half of participants (52.9%) stated that had faced at least one barrier in accessing healthcare or medication (Table 2):46.4% of the sample tackled at least one barrier in healthcare and 24.40% at least one barrier in medication. Missing values did not exceed 10% in any of the variables except for Fagerstrom total score (11.1%). Apart from Fagerstrom total score, the higher percentages of missing values were observed in the IPED (4.8%), HADS anxiety (7.6%) and HADS depression (10.4%) total scores and in the number of children (8.4%). 

It merits noting that the vast majority of the participants (92.8%) believed that were following their physician’s instructions to the fullest, whereas only 76% of participants was physician-rated to have a very good adherence. The correlation of measurements made on the same individual was found to be equal to 0.14 (95% C.I.: 0.02-0.26). The level of agreement between doctors and patients regarding treatment adherence was considered low. The correlation among mean ratings for each team of raters was 0.24 (95% C.I.: 0.04-0.41). 

Of great interest is the fact that 1 out of 4 patients reported smoking. According to the Fagerstrom scale a 41.1% of the smokers had a low to moderate dependence of the nicotine and a 37.5% a moderate dependence (Table 3).

Variables that were univariately associated with treatment adherence scores either rated on patients’ (Supplemental Table 1) or physicians’ perspective (Supplemental Table 2) at the p<0.10 level, were entered in a multiple ordinal logistic regression model. As a result, two different multiple ordinal logistic regressions were conducted.

In the first ordinal logistic regression model the condition of proportionality of odds is fulfilled (χ2=2.14, df=3, p-value>0.05). As shown in Table 4 none of the factors entered in the model was found to be independently associated with the dependent variable apart from having at least one barrier in access to medication. More specifically those patients that face at least one barrier in access to medication tend to have 2.82 times increased odds to report poorer adherence (or at most good) compared to those who have not faced any barrier. 

The proportionality of odds assumption was fulfilled for the second ordinal logistic regression as well (*χ*^2^=21.08, df=9, p-value>0.05). The factors that were found to be independently associated with the physicians’ rating on patients’ adherence were: the level of education, having at least one barrier in access to medication, the Fagerstrom total score and the HADS anxiety total score (Table 5). More specifically those patients who had fulfilled 12 years of education were 67% less likely to be rated as having bad/moderate (or at best good) adherence compared to those who had not completed 12 years of education. Furthermore, those patients who had higher education (defined as more than 12 years of education) had 72% less odds to be rated as having bad/moderate adherence (or at best good) compared to those at the reference category. Those patients who had faced at least one barrier in their access to medication were found to be 2.5 times more likely to be rated to have bad/moderate adherence (or at best good) compared with those who have not faced a barrier. Lastly a unit increase in the Fagerstrom scale score and in the HADS Anxiety subscale score was correlated with 41% and 15% increased odds to be rated as having poorer adherence by the physicians, respectively or at most good adherence by the assumptions of the proportional odds model.

**Table 1:** Sample’s Socio-Demographic and Clinical Characteristics (N=250)

Socio-demographic and clinical characteristics	
Gender (N, %)	
Male	175 (70.3)
Female	74 (29.7)
Citizenship(N, %)	
Other	8 (3.3)
Greek	238 (96.7)
Place of residence (N, %)	
Non-Athens	79 (32.4)
Athens	165 (67.6)
Age (mean, SD; years)^1^	65.15 (10.28)
Family status (N, %)	
Unmarried	19 (7.6)
Married-cohabits	189 (75.6)
Separated-Widowed	42 (16.8)
Children (N, %)	
No	26 (10.4)
Yes	224 (89.6)
Number of children (median, range)^2^	2 (1-6)
Educational level(N, %)	
<12 years	106 (42.4)
=12 years	92 (36.8)
>12 years	52 (20.8)
Employment status(N, %)	
Unemployed	23 (9.2)
Employed	55 (22.0)
Economicallyinactive	172 (68.8)
Health insurance(N, %)	
No	18 (7.2)
Yes	231 (92.8)
Total time without insurance (median, range; months)	60 (7-420)
Livingcondition (N, %)	
Alone	29 (11.7)
Withfamily	199 (79.6)
With other relative/non-relative	20 (8.0)
Cancertype(N, %)	
NSCLC	195 (80.9)
SCLC	46 (19.1)
Number of medicines (median, range)^3^	4 (0-16)
Total time since diagnosis (mean, SD; months)^4^	14.55 (17.34)
HADS anxiety subscale (N, %)	
Normal	141 (61.0)
Borderline	38 (16.5)
Abnormal	52 (22.5)
HADS depression subscale (N, %)	
Normal	110 (49.1)
Borderline	54 (24.1)
Abnormal	60 (26.8)

^1^Missing values, 2%; ^2^Missing values, 8.4%; ^3^Missing values, 2.8%; ^4^Missing values, 2.4%

**Table 2:** Barriers in Treatment and Socio-Economic Characteristics of the Sample (N=250)

Barriers in treatment and socio-economic characteristics	
Have faced at least one barrier (either in access to healthcare or in access to medication) (N, %)	
No	119 (47.6)
Yes	131 (52.4)
Have faced at least one barrier in access to health (N, %)	
No	134 (53.6)
Yes	116 (46.4)
Have faced at least one barrier in access to medication (N, %)	
No	189 (75.6)
Yes	61 (24.4)
Monthlyincome(N, %)	
0 – 600 euros	92 (37.9)
601 – 1200 euros	108 (44.4)
>1201 euros	43 (17.7)
IPED total score (mean, SD)^1^	9.11 (4.84)

^1^Missing values, 4.8%

**Table 3:** Descriptive Statistics of Treatment Adherence Measures (N=250)

Adherencemeasures	
Self-rated treatment adherence (N, %)	
Verygood	231 (92.8)
Good	15 (6.0)
Moderate/Bad	3 (1.2)
Therapists’ rating of patient treatment adherence (N, %)	
Verygood	186 (76.9)
Good	26 (10.7)
Moderate/Bad	30 (12.4)
Smoking (N, %; last 12 months)	
No	184 (74.5)
Yes	63 (25.5)
Total time since smoking cessation (median, range; months)	24 (1-480)
Cigarettes/week before diagnosis (median, range)	40 (3-420)
Fagerstrom Scale	
Lowdependence (n,%)	11 (19.6)
Low to moderate dependence (n, %)	23 (41.1)
Moderatedependence (n,%)	21 (37.5)
High dependence (n, %)	1 (1.8)

**Table 4:** Multiple Ordinal Logistic Regression with Patients’ Rating of Treatment Adherence as the Dependent Variable (N=235)

LR χ2(3) =8.98, p-value<0.05, Pseudo R^2^= 0.0651		
Variable	OR	95% C.I.
Number of medicines	1.12	0.95-1.32
Barriers in medication (Yes)^1^	2.82	1.01-8.09
Fagerstrom total score	1.14	0.92-1.41

CI, Confidence interval; LR, Likelihood Ratio; OR, Odds ratio; ^1^Ref. category, No

**Table 5:** Multiple Ordinal Logistic Regression with Physicians’ Rating of Treatment Adherence as the Dependent Variable (N=193)

LR χ2(9) = 45.04, p-value<0.001, Pseudo R^2^= 0.1757		
Variable	OR	95% C.I.
Age	1.04	0.99-1.08
Education		
<12 years	Ref.	
=12 years	0.33	0.13-0.82
>12 years	0.28	0.08-0.96
Cancer type		
NSCL	Ref.	
SCLC	2.15	0.88-5.25
Barriers in medication (Yes)1	2.45	1.03-5.86
Fagerstrom total score	1.41	1.17-1.69
Total number of medicines	1.08	0.94-1.23
HADS Anxiety total score	1.15	1.03-1.30
HADS Depression total score	0.95	0.82-1.06

CI, Confidence interval; LR, Likelihood Ratio; OR, Odds ratio; ^1^Ref. category, No

## Discussion

Participants’ rate of treatment adherence was considered high from both perspectives. According to patients, only a slim minority rated their level of adherence as moderate or bad (1.2%); whereas from the physician’s perspective the corresponding percentage was equally low, albeit not as low as that of patients This is in stark contrast with evidence indicating that non-adherence is a major health concern among lung cancer patients (Goren and Bonaventura, 2013), as well as among patients with other illnesses (Brownell andCohen, 1995; Norman et al., 2000). The high rates of adherence among participants may be attributed to theself-report mode of assessing it (Stirrat et al., 2015) to the illness itself. This concurs with the view that treatment adherence is better among oncology patients, as compared to non-oncology patients, due to the grave risk involved if medication is not taken as prescribed (Osterberg and Blaschke, 2005; Haynes et al., 2002). Nonetheless, the low concordance between the patient’s perspective and that of the clinician hints towards a communication gap between the two, lending support to the view that optimal adherence is bound to a good therapeutic alliance (Stewart et al., 1999; Elliott et al., 2000; Ickovics andMeade,2002). Concomitantly, the high percentage of good adherence is largely inconsistent with the finding that one out of four participants reported smoking during the study period. As smoking habits in the present sample cannot be explained by nicotine dependence, this contradiction raises an important concern with respect to the conceptualization of good adherence and its communication to patients.

This concern becomes heightened in light of the clinical and ethical importance of patient empowerment and shared decision making in clinical practice. A growing body of research substantiates that patients who understand the importance and rationale behind a recommended treatment plan are more eager to adhere to this treatment (Dellande et al., 2004). This is in line with evidence suggesting positive treatment outcomes of patient empowerment (Williams et al., 1998; Broadstock andMichie, 2000)and a link with therapy adherence (Prigge et al., 2015). Nonetheless, the association between empowerment and therapy adherence warrants further investigation. While information search and knowledge development has been found to facilitate therapy adherence; this does not hold true for decision-participation (Prigge et al., 2015). In the same study, Prigge and colleagues corroborate the importance of considering disease type as a moderator variable between patient empowerment and therapy adherence. They argue that patient empowerment exerts no impact on treatment adherence among patients with severe diseases, such as breast cancer. This is in line with some evidence indicating that patients with cancer do not want to participate in decision-making concerning their treatment due to the responsibility involved (Degner and Sloan,1992). Nonetheless, shared-decision making does not allude to patients and physicians sharing equal responsibility for treatment decision. Rather, it is expected from physicians to discuss evidence-based information about a disease and its treatment and allow patients to decide on thedegree of responsibility they want to bear for any health decision (Politi et al., 2013). 

Among potential risk factors for poor adherence, barriers to medication emerged as the only statistically significant predictor in both measures of adherence- patient rated and physician rated. This finding sheds light on systemic determinants of poor adherence and the role played by aspects of the health care system (Ruddy et al., 2009). This is of sheer importance if one takes into consideration that the majority of studies stress the contribution of personal/individual factors in explaining non-adherence, thus largely overlooking the contribution of systemic factors (Ruddy et al., 2009). One out of two participants of the present sample reported facing substantial impediments to accessing healthcare or medication. This resonates with previous work into healthcare access barriers among patients with rheumatoid arthritis (HOPE I), multiple sclerosis (HOPE II) and cancer (HOPE III) in Greece amid the recession. With respect to the latter, the results of HOPE III corroborated grave barriers among cancer patients in their endeavor to seek treatment(Souliotis et al., 2015). Long waiting times, long physical distance from treatment providers and out of pocket costs were found to be conducive to treatment delay, which in turn impinges on health outcomes. Concomitantly, evidence indicated an adverse impact of the recession with healthcare access being worse in 2015 as compared to 2009. 

Findings pertaining to the different risk factors emerging as predictors of poor adherence between the physician and the patient measure lend additional credence to the claim that patients and clinicians display variations in their conceptualization and understanding of adherence. 

*Study Limitations*

This study is among the few stressing the importance of socio-economic and systemic determinants of treatment adherence in lung cancer and the only study to have investigated a potential influence of the recession. Its results may inform interventions as well as policy recommendations. Patients have been recruited from the main public health treatment provider for lung cancer patients, rendering its findings representative of all patients with lung cancer in Greece. 

This study was not without its limitations. As the focus of the paper had been to investigate the influence of socio-economic and psychological determinants of treatment adherence, as this has been a largely neglected aspect of existing literature, the particular study concentrated on patients receiving the same treatment pathway. Therefore, patients with other disease characteristics were not taken into consideration. It is highly likely that adherence levels may be lower in less severe stages of lung cancer and findings cannot be extrapolated to other types of cancer. Moreover, as treatment adherence is multifaceted, a number of important determinants may have not been taken into consideration, such as aspects of the therapeutic alliance or social support, among others. Furthermore, one may argue that self-report is not an adequate measure of treatment adherence (Jasti et al., 2006), especially among the proponents of the microelectronic monitoring system (MEMS) method. Nonetheless, growing evidence indicates that each method has its advantages and disadvantages and no method is deemed the gold standard (Wagner et al., 2001). Additionally, theoretical and empirical arguments bolster the use of self-report (Hays and Di Matteo, 1987; Di Matteo et al., 2003); especially when speed, efficiency and low-cost measures are prioritized, as it is often the case in clinical settings (Stirrat et al., 2015). Self-report, especially when assessed by a single-item may lead to overestimation of adherence levels (Stirrat et al., 2015); however, as the study was conducted in clinical settings, where treating physicians could not have been administered longer interview schedules, and patients -suffering from a debilitating illness- could not have been burdened by a lot of questions, we opted for a single self-reported item A follow-up study should explore adherence levels to the different aspects of physician recommendations (e.g. pharmaceutical regime, smoking, lifestyle changes, dietary changes, hospital visits, etc.)

*Clinical Implications*

Potential differences in the conceptualization of adherence among physicians and patients warrant further exploration, as they may create communication gaps between the two parties, jeopardizing in this way the therapeutic alliance and treatment outcome. At the same time, researchers and physicians should try to reach consensus with respect to the definition and characteristics of adherence: is optimal adherence limited to medication adherence alone or does it include other aspects of the treatment plan, such as exercising, cessation of smoking among others? Once some form of consensus is reached, interventions should target the physician-patient communication. Physicians should endeavour to increase patients’ knowledge about cancer and its treatment as well as the importance and consequences of optimal adherence. They should try to engage patients in their treatment plan, giving them enough space to decide on the degree of responsibility they want to bear with respect to any health decision. They should promote the establishment of a fruitful partnership. Empowering patients, providing them with simple and clear instructions and listening carefully to their barriers, concerns and anxieties should be prioritized. At the same time, policy recommendations and interventions should be geared towards tackling access barriers to healthcare and medication. 

Evidence from oncology patients demonstrates a different perspective between patients and clinicians with respect to treatment adherence, hinting towards a communication gap between them. Moreover, systemic determinants of poor adherence should not be overlooked, as they appear to introduce barriers to optimal care. 

## Author Contribution Statement

KS conceived the study and participated in design of the study and drafting of the manuscript. LEP participated in design of the study and results interpretation and had a major contribution in drafting of the manuscript. ME participated in results interpretation and drafting of the manuscript. AM contributed in data acquisition and drafting of the manuscript. SN contributed in data analysis, results interpretation and drafting of the manuscript. MS participated in results interpretation and drafting of the manuscript. DV revised the manuscript for critical intellectual content. AP revised the manuscript for critical intellectual content. KNS participated in study design, supervised data acquisition process and revised the manuscript for critical intellectual content. All authors have read and approved the final version of the manuscript.
